# Prominent amyloid plaque pathology and cerebral amyloid angiopathy in APP V717I (London) carrier – phenotypic variability in autosomal dominant Alzheimer’s disease

**DOI:** 10.1186/s40478-020-0891-3

**Published:** 2020-03-12

**Authors:** Grace M. Lloyd, Jorge A. Trejo-Lopez, Yuxing Xia, Karen N. McFarland, Sarah J. Lincoln, Nilüfer Ertekin-Taner, Benoit I. Giasson, Anthony T. Yachnis, Stefan Prokop

**Affiliations:** 1 Center for Translational Research in Neurodegenerative Disease, University of Florida, Gainesville, FL 32610 USA; 2 Department of Neuroscience, University of Florida, Gainesville, FL 32610 USA; 3 Department of Pathology, University of Florida, Gainesville, FL 32610 USA; 4 Department of Neurology, University of Florida, Gainesville, FL 32610 USA; 5 Department of Neuroscience, Mayo Clinic, Jacksonville, FL 32224 USA; 6 Department of Neurology, Mayo Clinic, Jacksonville, FL 32224 USA; 7 McKnight Brain Institute, University of Florida, Gainesville, FL 32610 USA; 8 Fixel Institute for Neurological Diseases, University of Florida, Gainesville, FL 32610 USA

**Keywords:** Alzheimer’s disease, Amyloid precursor protein, Beta-amyloid, Cerebral amyloid angiopathy, London mutation, APOE

## Abstract

The discovery of mutations associated with familial forms of Alzheimer’s disease (AD), has brought imperative insights into basic mechanisms of disease pathogenesis and progression and has allowed researchers to create animal models that assist in the elucidation of the molecular pathways and development of therapeutic interventions. Position 717 in the amyloid precursor protein (APP) is a hotspot for mutations associated with autosomal dominant AD (ADAD) and the valine to isoleucine amino acid substitution (V717I) at this position was among the first ADAD mutations identified, spearheading the formulation of the amyloid cascade hypothesis of AD pathogenesis. While this mutation is well described in multiple kindreds and has served as the basis for the generation of widely used animal models of disease, neuropathologic data on patients carrying this mutation are scarce. Here we present the detailed clinical and neuropathologic characterization of an APP V717I carrier, which reveals important novel insights into the phenotypic variability of ADAD cases. While age at onset, clinical presentation and widespread parenchymal beta-amyloid (Aβ) deposition are in line with previous reports, our case also shows widespread and severe cerebral amyloid angiopathy (CAA). This patient also presented with TDP-43 pathology in the hippocampus and amygdala, consistent with limbic predominant age-related TDP-43 proteinopathy (LATE). The APOE ε2/ε3 genotype may have been a major driver of the prominent vascular pathology seen in our case. These findings highlight the importance of neuropathologic examinations of genetically determined AD cases and demonstrate striking phenotypic variability in ADAD cases.

## Introduction

Alzheimer’s disease (AD) is the most common form of dementia, currently affecting more than 5 million people in the United States [6]. Neuropathological hallmarks of AD include extracellular deposits of beta-Amyloid (Aβ), intracellular deposits of neurofibrillary tangles (NFT) and neuron loss [35]. The majority of cases occurs as sporadic disease (sporadic AD, SAD), modified by genetic, behavioral and environmental risk factors, while a subset of cases is caused by autosomal-dominant mutations [14, 21, 35]. These mutations in autosomal dominant forms of AD (ADAD) are clustered in genes associated with the metabolism of Aβ-peptides, which are generated from Amyloid Precursor Protein (APP) in sequential cleavage events mediated by β- and γ-secretase [9]. ADAD associated mutations in APP mainly cluster around these secretase cleavage sites, while codon 717 is a mutational hotspot at the γ-secretase cleavage site [27]. To date, four different pathogenic amino acid changes for APP on codon 717 have been described: Valine to Phenylalanine (V717F, Indiana [37]), Valine to Glycine (V717G [10]), Valine to Isoleucine (V717I, London [18, 63]) and Valine to Leucine (V717L [38]). All of these mutations shift the ratio of Aβ_1–42_ /Aβ_1–40_ towards increased production of Aβ_1–42_ [27], which is more aggregation prone and can drive pathological protein accumulation. The V717I (London) mutation, was among the first mutations described to cause ADAD and this discovery has put Aβ center stage in AD pathogenesis. Animal models overexpressing mutant human APP are a staple of AD research [17, 24, 46, 47, 49, 52, 54] and the APP V717I mutation was used to generate some widely used models [34, 43, 54]. Despite the numerous and detailed descriptions of pathological findings in these animal models, neuropathological characterization of patients carrying the APP V717I mutation is scarce. The brain of the original case from England was reported to show AD neuropathological changes with mild cerebral amyloid angiopathy (CAA) as well as Lewy body (LB) pathology in cortical and brainstem regions, while findings from an American family with this mutation showed AD pathology but no CAA or LB [8, 22, 36].

## Case presentation

The patient was a sixty-six (66) year-old right-handed Caucasian female with a past medical history of thyroid disease. Her family history was notable for extensive AD, involving her mother (deceased from disease at age 62), two aunts (deceased from disease at ages 66 and 68), and a grandfather (also deceased from disease). Additionally, she had a brother diagnosed with AD, still living in a nursing facility.

She was first noted to develop neurologic symptoms in her mid-fifties, manifesting primarily as progressive memory loss. She presented for formal neurologic evaluation at age 60, at which time her husband described significant memory difficulty, confusion, and occasional difficulty in finishing sentences. While she had discontinued working five years prior to evaluation due to difficulty with completing occupational tasks, she maintained her ability to finish routine housework. On initial evaluation, she was oriented to person, place, and time but not year, with a Mini Mental Status Exam (MMSE) score of 17. Physical exam findings included the presence of a tremor of her head and bilateral hands, with a negative Romberg’s test. These findings were assessed to be consistent with AD of moderate intensity, and she was started on donepezil and memantine therapy. Concurrent computed tomography (CT) imaging of her brain demonstrated subtle areas of low-attenuation in the periventricular white matter of the parietal lobe, suggestive of microvascular ischemic change.

Two years after initial diagnosis, she was noted to demonstrate significant clinical deterioration during a follow-up clinical visit. Her husband described a loss of her ability to maintain independent activities of daily living, becoming dependent on him for bathing and dressing on a daily basis, and that she had additionally developed urinary incontinence. Her recent medical history was notable for a hospital admission due to dehydration and severe hypothyroidism secondary to thyroid medication non-compliance. On evaluation, she exhibited depression, anxiety, confusion, and an MMSE score of 8. These findings were assessed to be consistent with a progression to severe AD. CT imaging at this time revealed mild global brain parenchymal loss, with no evidence of focal lesions or asymmetric atrophy.

Her symptoms continued to progress, whereupon at a follow-up visit four years after initial diagnosis, her husband described the development of intermittent jerking movements by the patient, occurring a few times per week and lasting approximately 15 min in duration. At this visit, she was oriented only to person, and her MMSE score was 0, because she was unable to follow commands. A subsequent electroencephalogram revealed no focal abnormalities or epileptiform activity, noting only the presence of diffuse slowing activity, consistent with moderate encephalopathy.

By the time of her final follow-up visit approximately, six years after initial diagnosis, she had developed a wide-based gait with frequent falls, aphasia, personality changes, poor insight, and complete lack of orientation to time, place, and person. She ultimately passed away within six months of her last visit, at age 66. Her family consented to neuropathologic evaluation of her brain by the Center for Translational Research in Neurodegenerative Disease (CTRND) at the University of Florida.

### Molecular studies

**Fig. 1:**
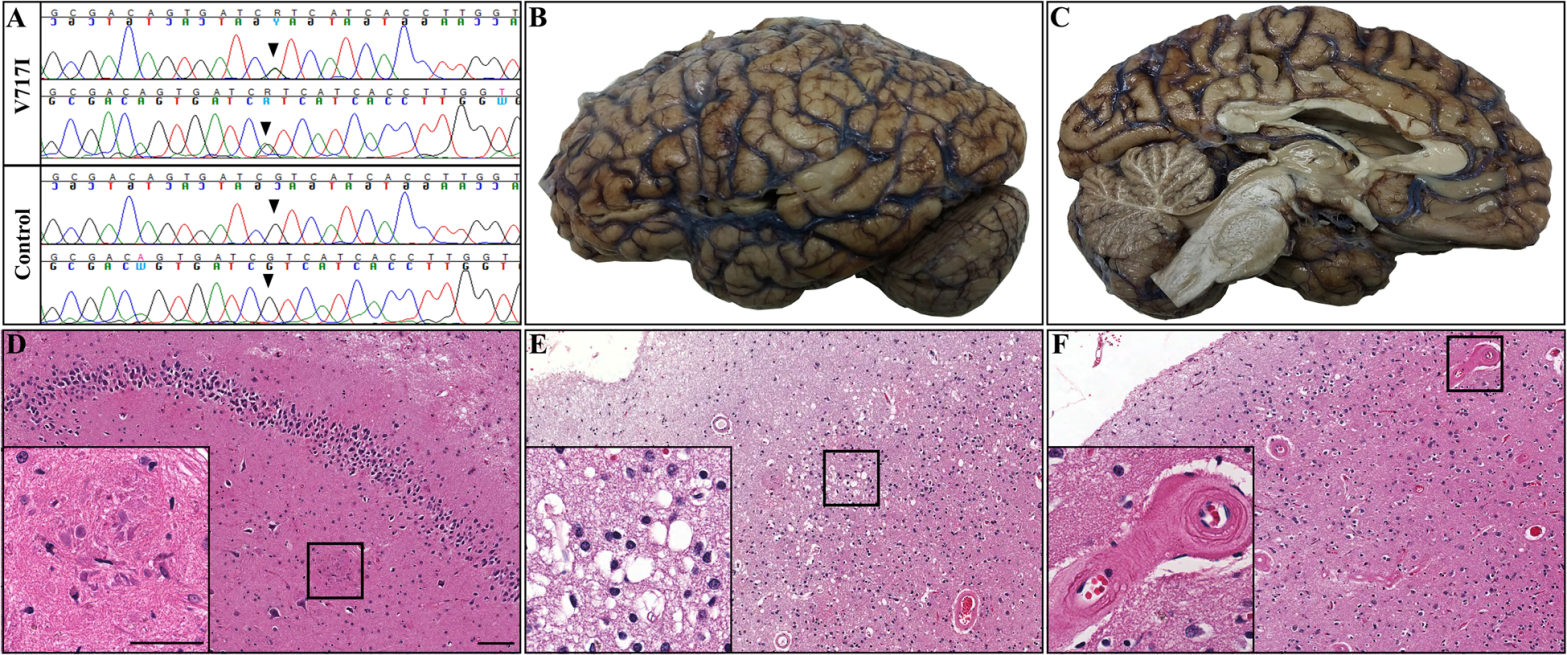
(**a**) Representative chromatogram of Sanger-sequencing revealed a guanine-to-adenine single nucleotide substitution at codon 717 of APP, resulting in a Valine to Isoleucine amino acid change in the ADAD patient (APP NM_000484.3 c2149G > A pVal717Ile). (**b, c**) Representative gross images of formalin-fixed left hemibrain. (**d - f**) Representative overview (scale bar = 2000 μm) and high magnification (insert, scale bar = 50 μm) images of H&E stained sections reveal neuron loss, astrogliosis and numerous neuritic plaques (**d**), superficial spongiosis (**e**), as well as substantial amyloid angiopathy of superficial cortical and leptomeningeal vessels (**f**)

A directed Sanger Sequencing screening panel for autosomal dominant mutations of *APP, PSEN1*, and *PSEN2* identified a guanine-to-adenine single nucleotide substitution at codon 717, resulting in a Valine to Isoleucine amino acid change (APP NM_000484.3 c2149G > A pVal717Ile, Fig. 1a). A PCR-based molecular assay for the *APOE* gene revealed the patient’s genotype to be ε2/ε3 (for details see Additional file 1).

### Neuropathological evaluation

The post-mortem interval prior to brain procurement was eight hours, with a fresh brain weight of 1080 g (for details see Additional file 1). Diffuse cerebral atrophy, with relative preservation of the cerebellum (Fig. 1b, c) was noted. Minimal to no atherosclerotic changes associated with the basal vasculature were identified. Serial coronal sections of cerebral hemispheres confirmed a mild to moderate degree of atrophy, with blunting of the lateral angles of the ventricles and sulcal widening most appreciable along the Sylvian fissure. No focal lesions were otherwise observed in the remainder of the cerebrum, brain stem, or cerebellum.

Microscopic examination demonstrated extensive neuronal loss and associated gliosis in the hippocampus and neocortical areas, with numerous pyramidal neurons notable for flame-shaped neurofibrillary tangles. Multiple areas demonstrated prominent neuritic plaques (Fig. 1d, insert) with associated areas of neuronal loss, gliosis, and variable vacuolization and spongiosis of superficial cortical layers (Fig. 1e). In addition, extensive cerebral amyloid angiopathy (CAA) was apparent throughout parts of cerebrum and cerebellum, concentrating on superficial cortical and leptomeningeal blood vessels (Fig. 1f). In contrast, only mild small vessel hyalinization of the basal ganglia and white matter were identified, with focal calcification of globus pallidus blood vessels. A remote microhemorrhage was identified in the primary sensory cortex. The brainstem and cerebellum demonstrated no major neuropathologic changes, with no significant neuronal loss, gliosis, or Lewy bodies identified in the substantia nigra or locus coeruleus.

**Fig. 2:**
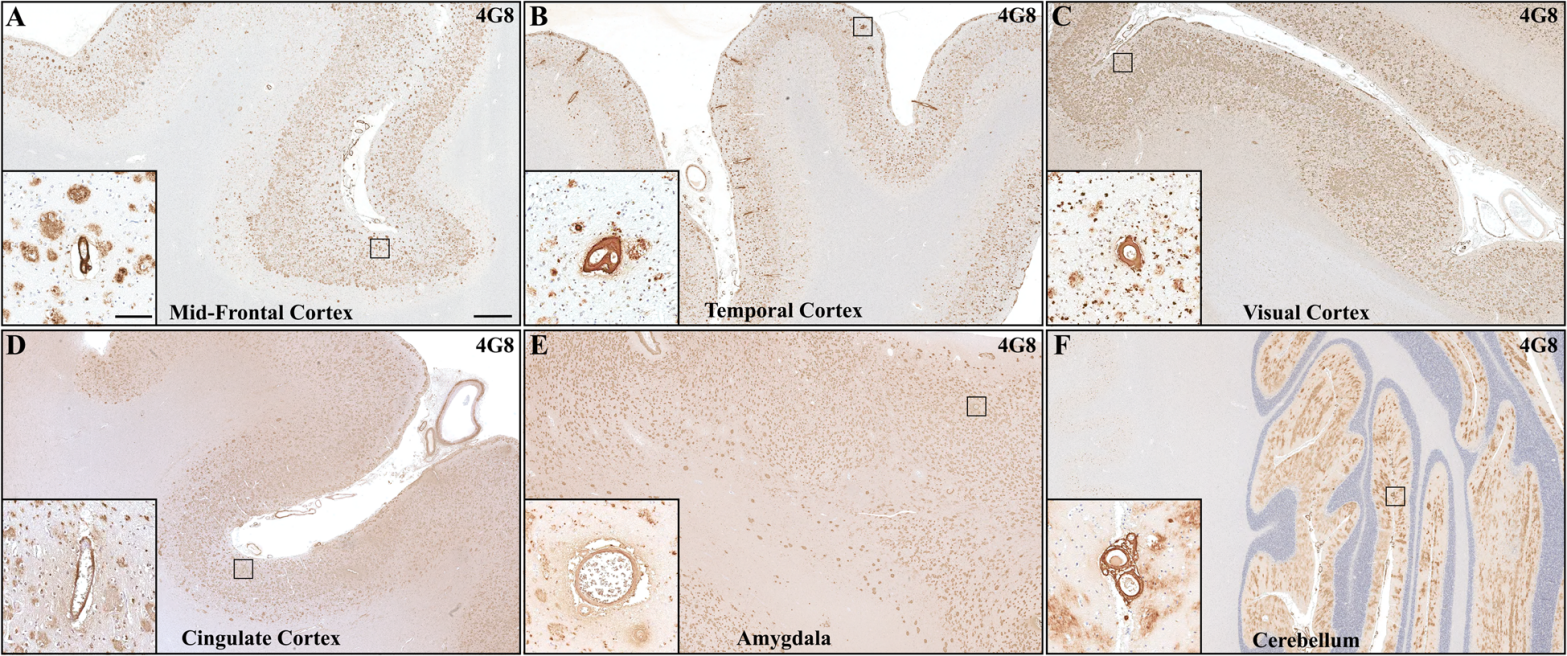
Representative images of 4G8-stained sections of mid-frontal cortex (**a**), superior temporal gyrus (**b**), visual cortex (**c**), cingulate gyrus (**d**), amygdala (**e**) and cerebellum (**f**) reveal widespread parenchymal and vascular (inserts) Aβ-pathology throughout the neuroaxis. Cerebral amyloid angiopathy was more prominent in cortical and leptomeningeal vasculature with no substantial involvement of cortical capillaries. Overview images (scale bar = 1000 μm) and high magnification insert (scale bar = 50 μm)

**Fig. 3:**
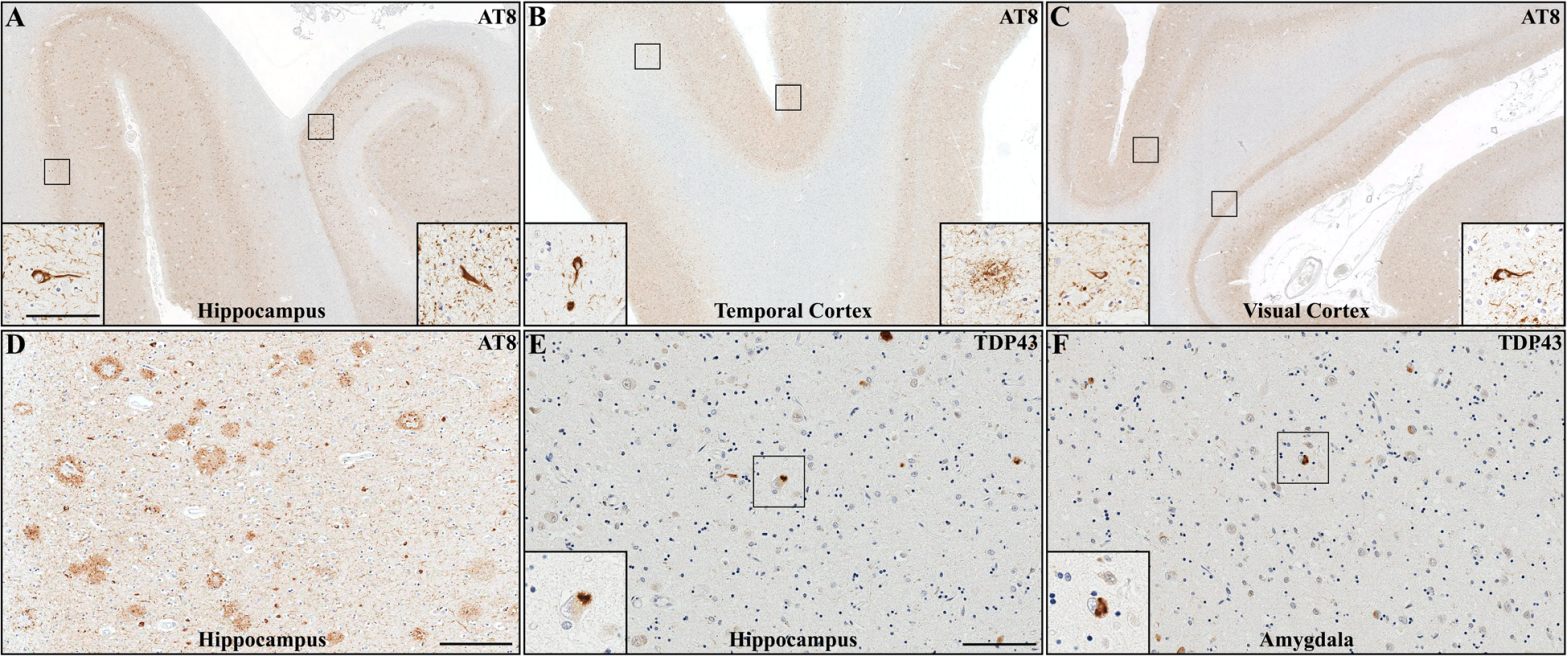
Representative overview (scale bar = 2000 μm) and high magnification (insert, scale bar = 50 μm) images of AT8 staining in APP V717I mutation carrier for the hippocampus (**a**), superior temporal gyrus (**b**) and visual cortex (**c**) reveal substantial NFT pathology in all regions examined. (**d**) AT8 staining also reveals “frequent” neuritic plaques in the inferior temporal cortex (scale bar = 1000 μm, insert scale bar = 50 μm). Representative overview (scale bar = 2000 μm) and high magnification (insert, scale bar = 50 μm) images of TDP-43 staining demonstrate TDP-43 positive inclusions in hippocampus (**e**) and amygdala (**f**)

Immunohistochemistry (for details see Additional files 1 and 2) with a pan-Aβ antibody (4G8) demonstrated a very high Aβ plaque burden throughout the cerebral neocortex (Fig. 2a-d), the amygdala (Fig. 2e), basal ganglia, and tegmentum of the midbrain and pontine brainstem. Aβ deposition was also identified in the cerebellum, presenting as scattered fleecy diffuse plaques and neuritic plaques in the molecular layer of the cerebellar cortex (Fig. 2f). These findings translated to Thal phase 5 of Aβ deposition, corresponding to an “A3” plaque score according to the 2012 NIA-AA criteria [35]. The majority of Aβ plaques were surrounded by dystrophic neurites (Fig. 3d), with the frequency of neuritic plaques throughout the neuroaxis corresponding to a CERAD semiquantitative score of “frequent” (C3) [35].

Immunostaining for tau demonstrated a concordant pattern of severe, widespread inclusion pathology, manifesting as a heavy burden of intraneuronal neurofibrillary tangles (NFT) and dystrophic neurites associated with amyloid plaques (Fig. 3a-d). Disease topography extended from the entorhinal cortex and adjacent mesial temporal lobe cortex, deep cerebral gray matter structures, to several areas of neocortex including primary visual cortex (Fig. 3a-c). These findings correspond to an advanced Braak stage (VI), translating to a “B3” NFT score [35].

In addition, intracytoplasmic neuronal inclusions were highlighted in the hippocampal subiculum and amygdala by immunohistochemistry for TDP-43 (Fig. 3e-f), while neocortical areas were devoid of TDP-43 pathology, corresponding to stage 2 of the recently defined limbic-predominant age-related TDP-43 encephalopathy neuropathologic change (LATE-NC [41]). No α-synuclein immunoreactive pathology was identified in the examined sections of cerebrum, brainstem, or cerebellum (data not shown).

**Fig. 4:**
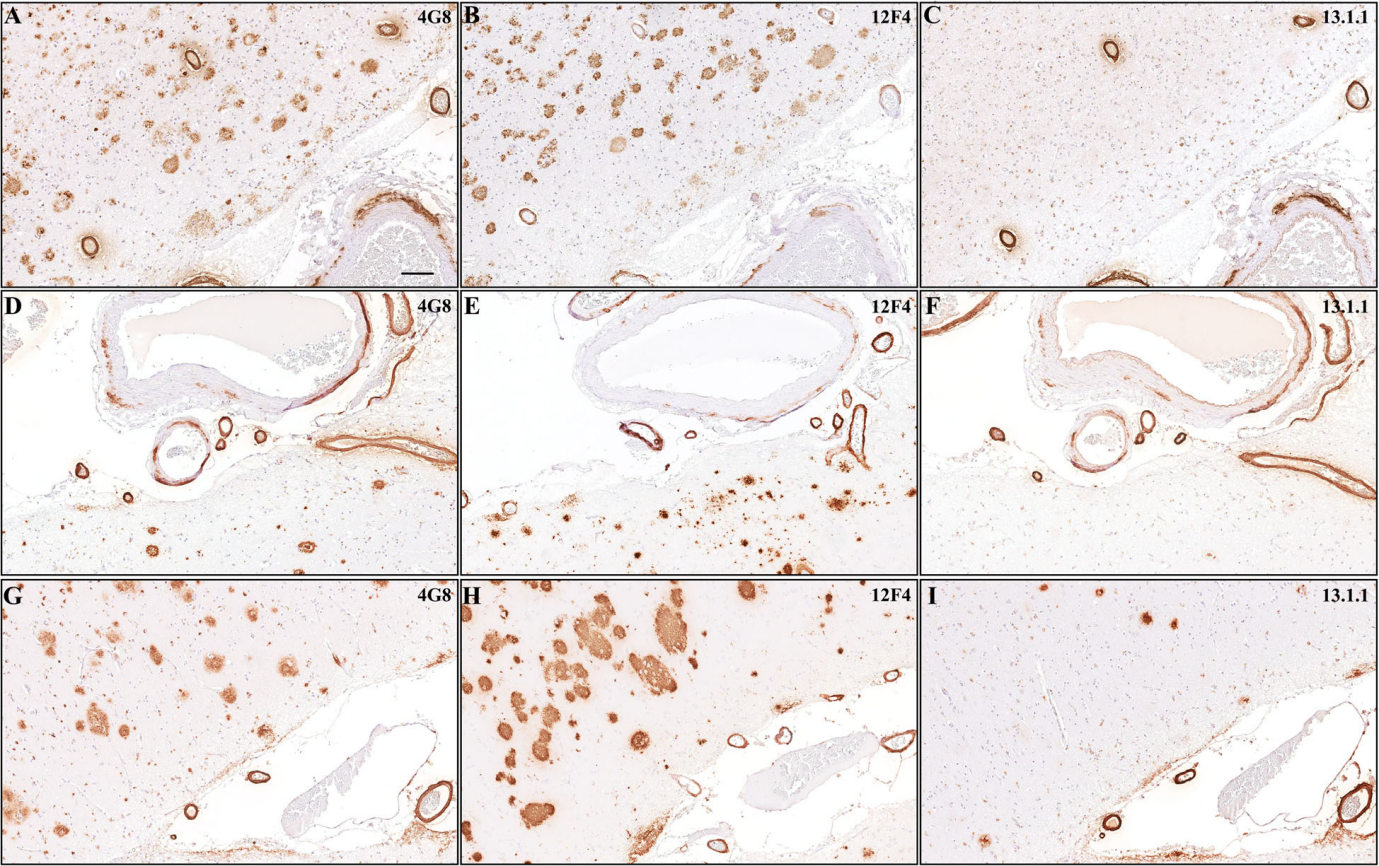
(**a - c**) Representative images of superior temporal cortex sections of APP V717I mutation carrier labelled with pan-Aβ (4G8, **a**) antibodies, demonstrate strong labeling of parenchymal and vascular amyloid deposits. Vascular deposits also showed strong staining with Aβ_1–42_ specific antibodies (12F4, **b**) and Aβ_1–40_ specific antibodies (13.1.1, **c**). Parenchymal amyloid deposits demonstrated strong Aβ_1–42_ positivity (12F4, **b**), while being scarcely labelled with Aβ_1–40_ specific antibodies (13.1.1, **c**). Vascular amyloid in two SAD cases with different APOE genotype (ɛ3/ɛ3 (**d-f**) and ɛ4/ɛ4 (**g-i**)), showed a similar staining pattern with strong positivity for pan-Aβ antibodies (4G8, **d, g**), as well as Aβ_1–42_ (**e, h**) and Aβ_1__− 40_ specific antibodies (**f, i**). Parenchymal amyloid in SAD cases were highlighted with pan-Aβ (**d, g**) and Aβ_1–42_ specific antibodies (**e, h**), and showed some reactivity with Aβ_1–40_ specific antibodies (**f, i**). Overview images (scale bar = 100 μm)

Aβ pathology was additionally noted to manifest as severe, widespread cerebral amyloid angiopathy (CAA), with a predilection for the superficial cortical and leptomeningeal vasculature and relative sparing of cortical capillaries (CAA type 2, [58]). Prominent vascular Aβ-deposits were detected in multiple neocortical areas (Fig. 2a-c), limbic areas (Fig. 2d-e) and the cerebellum (Fig. 2f), while sections from thalamus, basal ganglia, pons and medulla did not show vascular Aβ-deposits. This corresponds to CAA stage 2 according to Thal et al. [57]. Focal double barreling (Fig. 2b) and disruption of vessel wall integrity was noted, corresponding to severe CAA [61] or grade 4 CAA [44]. Vascular Aβ deposits showed strong immunoreactivity with pan-Aβ antibodies (4G8, Fig. 4a), and were labelled with Aβ_1–42_ specific antibodies (12F4, Fig. 4b), as well as Aβ_1–40_ specific antibodies (13.1.1 [31], Fig. 4c). In contrast, parenchymal Aβ plaques were labelled strongly with pan-Aβ (Fig. 4a) and Aβ_1–42_ specific (Fig. 4b) antibodies, while Aβ_1–40_ specific antibodies only stained a minority of Aβ-plaques (Fig. 4c). We contrasted these results with staining in two cases of SAD with different APOE genotype (APOE ε3/ε3 and APOE ε4/ε4, for details see Additional file 3: Table S2). While CAA in both SAD cases showed a similar pattern of immunoreactivity with pan-Aβ antibodies (Fig. 4d, g), as well as Aβ_1–42_ (Fig. 4e, h) and Aβ_1–40_ specific antibodies (Fig. 4f, i) compared to our ADAD case (Fig. 4 A-C), parenchymal deposits were highlighted to a much greater extent with Aβ_1–40_ antibodies (Fig. 4F, I) than in our ADAD case (Fig. 4c).

## Discussion and conclusions

The identification of missense mutations in APP underlying familial forms of AD has paved the way for the formulation of the “amyloid cascade hypothesis” [16, 22] by placing the generation of Aβ peptides as central to disease pathophysiology. To date, more than 50 mutations in APP associated with early onset AD have been described [9, 16, 29, 50, 55]; rare APP variants associated with protective properties have also been reported [28]. Several different substitutions of the intramembranous Valine residue at position 717 of APP have been described to be associated with familial forms of AD [10, 18, 37, 38]. This position is near the γ-secretase cleavage site of APP, such that amino acid substitutions at this functional locus lead to an increased ratio of Aβ_1–42_/Aβ_1–40_ with a trend towards increased production of Aβ_1–42_ [15, 27, 49].

The patient described here carrying the APP V717I mutation presented with extensive AD neuropathological changes, including abundant and widespread Aβ-pathology in cerebrum, subcortical nuclei, and cerebellum. Multiple different types of plaques were noted, including core-plaques, diffuse plaques and subpial band-like Aβ deposits. All of the deposits showed a uniformly strong staining pattern with Aβ_1–42_ specific antibodies and relative scarcity of Aβ_1–40_ positivity compared to SAD cases, in line with the reported increase in Aβ_1–42_ with this mutation in cell culture studies [27]. The majority of Aβ-deposits were associated with dystrophic neurites containing phosphorylated tau species. In addition, widespread neuronal tau pathology in the form of NFT, as well as neuropil thread pathology were noted. While no concomitant α-synuclein pathology was detected, TDP-43 positive inclusions were observed in the amygdala and hippocampus. Co-occurring TDP-43 pathology in carriers of APP mutations is not as common as in sporadic (late-onset) AD but has been reported [11]. This is in line with a contribution of age to the preponderance of TDP-43 positive pathology and ties in with the recently proposed entity of limbic-predominant TDP-43 neuropathological changes (LATE-NC) [41]. In addition, severe and widespread CAA was noted, affecting leptomeningeal and cortical blood vessels, but sparing fine capillaries. Systematic studies on CAA in ADAD cases are scarce, but a recent report from the National Alzheimer Coordinating Center (NACC) showed an increased CAA score in ADAD compared to SAD [48]. The number of APP mutation carriers in this study were limited, but phenotypic variability with respect to CAA severity was observed. Vascular amyloid in our ADAD case was strongly labelled with pan-Aβ, as well as Aβ_1–42_ and Aβ_1–40_ specific antibodies in a similar pattern as observed in two SAD cases with different APOE genotype. The relative abundance of Aβ species in parenchymal and vascular deposits has been a matter of intense debate. Initial reports suggested that the majority of vascular Aβ is Aβ_1–40_ [1, 19, 25, 26, 45], but subsequently a substantial contribution of Aβ_1–42_ to vascular Aβ-deposition was acknowledged [1, 19, 40, 51, 60], with some reports suggesting Aβ_1–42_ deposition driving more severe CAA [4, 20]. Mechanistic studies in murine models indicate that initial deposition of Aβ_1–42_ may be necessary to drive subsequent Aβ_1–40_ deposition in blood vessels [33, 42], but the impact of different APOE genotypes has not been analyzed systematically in this context. The relative sparing of capillary vessels by pathologic Aβ deposits, referred to as type 2 CAA [58] was previously reported to be associated with the presence of at least one *APOE* ε2 allele [2, 32, 59]. *APOE* is currently the strongest known genetic risk factor of AD, with the ε4 isoform correlating to an increased incidence of AD in people of European descent [56]. The ε3 allele is associated with preservation of synaptic integrity in old human APP (hAPP) mice [7], and mediation of amyloid clearance in comparison to ε4 [5], but it was also correlated to an earlier age of onset than ε4 in this model [7, 30]. The ε2 genotype was studied in several Italian families with the *APP* V717I mutation. It was discovered that this allele was associated with a delayed age of onset compared to individuals with the same *APP* mutation but *APOE* ε3 homozygotes or ε4 carriers [23, 39, 53]. Furthermore, a recent report demonstrated protective effects of the Christchurch *APOE* variant in a carrier of the Presenilin (*PSEN*) E280A mutation [3]. These disease-modifying effects of the *APOE* genotype may provide one possible explanation for the divergent phenotypes seen in the clinical and neuropathological presentation of ADAD.

The case presented herein underscores the importance of neuropathological characterization of genetically determined cases of AD. Such examinations serve to identify phenotypic diversity within the disease, clarify potential modifiers of disease progression (such as, but no limited to, APOE genotype), explore the complex interrelations between disease mechanisms, and ultimately aid in elucidating potential therapeutic targets.

## Supplementary information

**Additional file 1.** Materials and Methods [12, 13, 31, 35, 62]

**Additional file 2: Table S1.** List of Antibodies used for this study

**Additional file 3:Table S2.** Summary of patient samples used in this study [35, 41, 61]

## Data Availability

All data generated or analyzed during this study are included in this published article.

## Abbreviations

ABC: Avidin-biotin complex
AD: Alzheimer’s disease
ADAD: Autosomal dominant AD
APOE: Apolipoproteins-E
APP: Amyloid precursor protein
Aβ: Beta-Amyloid
CAA: Cerebral amyloid angiopathy
CT: Computed tomography
CTRND: Center for Translational Research in Neurodegenerative Disease
DAB: 3,3′-diaminobenzidine
FAD: Familial Alzheimer’s disease
FBS: Fetal bovine serum
hAPP: Human amyloid precursor protein
HIER: Heat-induced epitope retrieval
LATE-NC: Limbic-predominant TDP-43 neuropathological changes
MMSE: Mini Mental Status Exam
NFT: Neurofibrillary tangles
PBS: Phosphate buffered saline
PS1: Presenilin 1
PS2: Presenilin 2
RT: Room temperature
SAD: Sporadic Alzheimer’s disease
UF HBTB: University of Florida Neuromedicine Human Brain Tissue Bank

## References

[1] Akiyama H, Mori H, Sahara N, Kondo H, Ikeda K, Nishimura T, Oda T, McGeer PL (1997). Variable deposition of amyloid β-protein (Aβ) with the carboxy- terminus that ends at residue valine40 (Aβ40) in the cerebral cortex of patients with Alzheimer’s disease: a double-labeling immunohistochemical study with antibodies. Neurochem Res.

[2] Allen N, Robinson AC, Snowden J, Davidson YS, Mann DMA (2014). Patterns of cerebral amyloid angiopathy define histopathological phenotypes in Alzheimer’s disease. Neuropathol Appl Neurobiol.

[3] Arboleda-Velasquez JF, Lopera F, O’Hare M, Delgado-Tirado S, Marino C, Chmielewska N, Saez-Torres KL, Amarnani D, Schultz AP, Sperling RA, Leyton-Cifuentes D, Chen K, Baena A, Aguillon D, Rios-Romenets S, Giraldo M, Guzmán-Vélez E, Norton DJ, Pardilla-Delgado E, Artola A, Sanchez JS, Acosta-Uribe J, Lalli M, Kosik KS, Huentelman MJ, Zetterberg H, Blennow K, Reiman RA, Luo J, Chen Y, Thiyyagura P, Su Y, Jun GR, Naymik M, Gai X, Bootwalla M, Ji J, Shen L, Miller JB, Kim LA, Tariot PN, Johnson KA, Reiman EM, Quiroz YT (2019) Resistance to autosomal dominant Alzheimer’s disease in an APOE3 Christchurch homozygote: a case report. Nat Med 25. 10.1038/s41591-019-0611-3 10.1038/s41591-019-0611-3 PMC6898984 31686034

[4] Attems J, Lintner F, Jellinger KA (2004). Amyloid β peptide 1-42 highly correlates with capillary cerebral amyloid angiopathy and Alzheimer disease pathology. Acta Neuropathol.

[5] Beffert U, Aumont N, Dea D, Lussier-Cacan S, Davignon J, Poirier J (1999). Apolipoprotein E isoform-specific reduction of extracellular amyloid in neuronal cultures. Brain Res Mol Brain Res.

[6] Brookmeyer R, Abdalla N, Kawas CH, Corrada MM (2018). Forecasting the prevalence of preclinical and clinical Alzheimer’s disease in the United States. Alzheimers Dement.

[7] Buttini M, Yu GQ, Shockley K, Huang Y, Jones B, Masliah E, Mallory M, Yeo T, Longo FM, Mucke L (2002). Modulation of Alzheimer-like synaptic and cholinergic deficits in transgenic mice by human apolipoprotein E depends on isoform, aging, and overexpression of amyloid β peptides but not on plaque formation. J Neurosci.

[8] Cairns NJ, Chadwick A, Lantos PL, Levy R, Rossor MN (1993). Beta A4 protein deposition in familial Alzheimer’s disease with the mutation in codon 717 of the beta A4 amyloid precursor protein gene and sporadic Alzheimer’s disease. Neurosci Lett.

[9] Carmona S, Hardy J, Guerreiro R (2018). The genetic landscape of Alzheimer disease. Handb Clin Neurol.

[10] Chartier-Harlin MC, Crawford F, Houlden H, Warren A, Hughes D, Fidani L, Goate A, Rossor M, Roques P, Hardy J (1991). Early-onset Alzheimer’s disease caused by mutations at codon 717 of the beta-amyloid precursor protein gene. Nature.

[11] Davidson YS, Raby S, Foulds PG, Robinson A, Thompson JC, Sikkink S, Yusuf I, Amin H, DuPlessis D, Troakes C, Al-Sarraj S, Sloan C, Esiri MM, Prasher VP, Allsop D, Neary D, Pickering-Brown SM, Snowden JS, Mann DM (2011). TDP-43 pathological changes in early onset familial and sporadic Alzheimer’s disease, late onset Alzheimer’s disease and Down’s syndrome: association with age, hippocampal sclerosis and clinical phenotype. Acta Neuropathol.

[12] Dhillon J-KS, Riffe C, Moore BD, Ran Y, Chakrabarty P, Golde TE, Giasson BI (2017). A novel panel of α-synuclein antibodies reveal distinctive staining profiles in synucleinopathies. PLoS One.

[13] Duda JE, Giasson BI, Gur TL, Montine TJ, Robertson D, Biaggioni I, Hurtig HI, Stern MB, Gollomp SM, Grossman M, Lee VM, Trojanowski JQ (2000). Immunohistochemical and biochemical studies demonstrate a distinct profile of alpha-synuclein permutations in multiple system atrophy. J Neuropathol Exp Neurol.

[14] Duyckaerts C, Potier MC, Delatour B (2008). Alzheimer disease models and human neuropathology: similarities and differences. Acta Neuropathol.

[15] Fagan AM, Watson M, Parsadanian M, Bales KR, Paul SM, Holtzman DM (2002). Human and murine ApoE markedly alters A beta metabolism before and after plaque formation in a mouse model of Alzheimer’s disease. Neurobiol Dis.

[16] Finckh U, Kuschel C, Anagnostouli M, Patsouris E, Pantes GV, Gatzonis S, Kapaki E, Davaki P, Lamszus K, Stavrou D, Gal A (2005). Novel mutations and repeated findings of mutations in familial Alzheimer disease. Neurogenetics.

[17] Games D, Adams D, Alessandrini R, Barbour R, Borthelette P, Blackwell C, Carr T, Clemens J, Donaldson T, Gillespie F, Guido T, Hagopian S, Johnson-Wood K, Khan K, Lee M, Leibowitz P, Lieberburg I, Little S, Masliah E, Mc Conlogue L, Montoya-Zavala M, Mucke L, Paganini L, Penniman E, Power M, Schenk D, Seubert P, Snyder B, Soriano F, Tan H, Vitale J, Wadsworth S, Wolozin B, Zhao J (1995). Alzheimer-type neuropathology in transgenic mice overexpressing V717F β-amyloid precursor protein. Nature.

[18] Goate A, Chartier-Harlin MC, Mullan M, Brown J, Crawford F, Fidani L, Giuffra L, Haynes A, Irving N, James L (1991). Segregation of a missense mutation in the amyloid precursor protein gene with familial Alzheimer’s disease. Nature.

[19] Gravina SA, Ho L, Eckman CB, Long KE, Otvos L, Younkin LH, Suzuki N, Younkin SG (1995). Amyloid β protein (Aβ) in Alzheimer’s disease brain. Biochemical and immunocytochemical analysis with antibodies specific for forms ending at Aβ40 or Aβ42(43). J Biol Chem.

[20] Haglund M, Kalaria R, Slade JY, Englund E (2006). Differential deposition of amyloid β peptides in cerebral amyloid angiopathy associated with Alzheimer’s disease and vascular dementia. Acta Neuropathol.

[21] Hardy J, Mullan M, Chartier-Harlin MC, Brown J, Goate A, Rosso M, Collinge J, Roberts G, Luthert P, Lantos P, Naruse S, Kaneko K, Tsuji S, Miyatake T, Shimizu T, Kojima T, Nakano I, Yoshioka K, Sakaki Y, Miki T, Katsuya T, Ogihara T, Roses A, Pericak-Vance M, Haan J, Roos R, Lucotte G, David F (1991) Molecular classification of Alzheimer's disease. Lancet 337(8753):1342-1343. 10.1016/0140-6736(91)93011-W

[22] Hardy J (2017). The discovery of Alzheimer-causing mutations in the APP gene and the formulation of the “amyloid cascade hypothesis”. FEBS J.

[23] Holtzman DM, Bales KR, Tenkova T, Fagan AM, Parsadanian M, Sartorius LJ, Mackey B, Olney J, McKeel D, Wozniak D, Paul SM (2000). Apolipoprotein E isoform-dependent amyloid deposition and neuritic degeneration in a mouse model of Alzheimer’s disease. Proc Natl Acad Sci U S A.

[24] Hsiao K, Chapman P, Nilsen S, Eckman C, Harigaya Y, Younkin S, Yang F, Cole G (1996). Correlative memory deficits, Aβ elevation, and amyloid plaques in transgenic mice. Science (80- ).

[25] Iwatsubo T, Mann DMA, Odaka A, Suzuki N, Ihara Y (1995). Amyloid β protein (Aβ) deposition: Aβ42(43) precedes Aβ40 in down Syndrome. Ann Neurol.

[26] Joachim CL, Duffy LK, Morris JH, Selkoe DJ (1988). Protein chemical and immunocytochemical studies of meningovascular β-amyloid protein in Alzheimer’s disease and normal aging. Brain Res.

[27] De Jonghe C, Esselens C, Kumar-Singh S, Craessaerts K, Serneels S, Checler F, Annaert W, Van Broeckhoven C, De Strooper B (2001). Pathogenic APP mutations near the gamma-secretase cleavage site differentially affect Abeta secretion and APP C-terminal fragment stability. Hum Mol Genet.

[28] Jonsson T, Atwal JK, Steinberg S, Snaedal J, Jonsson PV, Bjornsson S, Stefansson H, Sulem P, Gudbjartsson D, Maloney J, Hoyte K, Gustafson A, Liu Y, Lu Y, Bhangale T, Graham RR, Huttenlocher J, Bjornsdottir G, Andreassen OA, Jönsson EG, Palotie A, Behrens TW, Magnusson OT, Kong A, Thorsteinsdottir U, Watts RJ, Stefansson K (2012). A mutation in APP protects against Alzheimer’s disease and age-related cognitive decline. Nature.

[29] Lantos PL, Luthert PJ, Hanger D, Anderton BH, Mullan M, Rossor M (1992). Familial Alzheimer’s disease with the amyloid precursor protein position 717 mutation and sporadic Alzheimer’s disease have the same cytoskeletal pathology. Neurosci Lett.

[30] Levi O, Michaelson DM (2007). Environmental enrichment stimulates neurogenesis in apolipoprotein E3 and neuronal apoptosis in apolipoprotein E4 transgenic mice. J Neurochem.

[31] Levites Y, Das P, Price RW, Rochette MJ, Kostura LA, McGowan EM, Murphy MP, Golde TE (2006). Anti-Abeta42- and anti-Abeta40-specific mAbs attenuate amyloid deposition in an Alzheimer disease mouse model. J Clin Invest.

[32] Mann DMA, Davidson YS, Robinson AC, Allen N, Hashimoto T, Richardson A, Jones M, Snowden JS, Pendleton N, Potier MC, Laquerrière A, Prasher V, Iwatsubo T, Strydom A (2018). Patterns and severity of vascular amyloid in Alzheimer’s disease associated with duplications and missense mutations in APP gene, Down syndrome and sporadic Alzheimer’s disease. Acta Neuropathol.

[33] McGowan E, Pickford F, Kim J, Onstead L, Eriksen J, Yu C, Skipper L, Murphy MP, Beard J, Das P, Jansen K, DeLucia M, Lin WL, Dolios G, Wang R, Eckman CB, Dickson DW, Hutton M, Hardy J, Golde T (2005). Aβ42 is essential for parenchymal and vascular amyloid deposition in mice. Neuron.

[34] Moechars D, Dewachter I, Lorent K, Reversé D, Baekelandt V, Naidu A, Tesseur I, Spittaels K, Haute CV, Checler F, Godaux E, Cordell B, Van Leuven F (1999). Early phenotypic changes in transgenic mice that overexpress different mutants of amyloid precursor protein in brain. J Biol Chem.

[35] Montine TJ, Phelps CH, Beach TG, Bigio EH, Cairns NJ, Dickson DW, Duyckaerts C, Frosch MP, Masliah E, Mirra SS, Nelson PT, Schneider JA, Thal DR, Trojanowski JQ, Vinters HV, Hyman BT, Aging NI on, Association A (2012). National Institute on Aging-Alzheimer’s Association guidelines for the neuropathologic assessment of Alzheimer’s disease: a practical approach. Acta Neuropathol.

[36] Mullan MJ, Giuffra L, Hardy JA, Ovenstone I, Haynes AR, James LA, Williamson R, Newton PJ, Owen MJ, Roques P, Luthert P, Lantos P, Goate AM, Rossor MN (1991) Clinical and Pathologic Features of Chromosome 21-Linked Familial Alzheimer's Disease. Ann NY Acad Sci 640(1):177-180. 10.1111/j.1749-6632.1991.tb00212.x 10.1111/j.1749-6632.1991.tb00212.x 1776736

[37] Murrell J, Farlow M, Ghetti B, Benson MD (1991). A mutation in the amyloid precursor protein associated with hereditary Alzheimer’s disease. Science (80- ).

[38] Murrell JR, Hake AM, Quaid KA, Farlow MR, Ghetti B (2000) Early-Onset Alzheimer Disease Caused by a New Mutation (V717L) in the Amyloid Precursor Protein Gene. Arch Neurol 57(6):885–887. 10.1001/archneur.57.6.885 10.1001/archneur.57.6.885 10867787

[39] Nacmias B, Latorraca S, Piersanti P, Forleo P, Piacentini S, Bracco L, Amaducci L, Sorbi S (1995). ApoE genotype and familial Alzheimer’s disease: a possible influence on age of onset in APP717 Val-->Ile mutated families. Neurosci Lett.

[40] Natté R, Yamaguchi H, Maat-Schieman MLC, Prins FA, Neeskens P, Roos RAC, Van Duinen SG (1999). Ultrastructural evidence of early non-fibrillar Aβ42 in the capillary basement membrane of patients with hereditary cerebral hemorrhage with amyloidosis, Dutch type. Acta Neuropathol.

[41] Nelson PT, Dickson DW, Trojanowski JQ, Jack CR, Boyle PA, Arfanakis K, Rademakers R, Alafuzoff I, Attems J, Brayne C, Coyle-Gilchrist ITS, Chui HC, Fardo DW, Flanagan ME, Halliday G, Hokkanen SRK, Hunter S, Jicha GA, Katsumata Y, Kawas CH, Keene CD, Kovacs GG, Kukull WA, Levey AI, Makkinejad N, Montine TJ, Murayama S, Murray ME, Nag S, Rissman RA, Seeley WW, Sperling RA, White Iii CL, Yu L, Schneider JA (2019). Limbic-predominant age-related TDP-43 encephalopathy (LATE): consensus working group report. Brain.

[42] Nishitsuji K, Tomiyama T, Ishibashi K, Kametani F, Ozawa K, Okada R, Maat-Schieman ML, Roos RAC, Iwai K, Mori H (2007). Cerebral vascular accumulation of Dutch-type Abeta42, but not wild-type Abeta42, in hereditary cerebral hemorrhage with amyloidosis, Dutch type. J Neurosci Res.

[43] Oakley H, Cole SL, Logan S, Maus E, Shao P, Craft J, Guillozet-Bongaarts A, Ohno M, Disterhoft J, Van Eldik L, Berry R, Vassar R (2006). Intraneuronal β-amyloid aggregates, neurodegeneration, and neuron loss in transgenic mice with five familial Alzheimer’s disease mutations: potential factors in amyloid plaque formation. J Neurosci.

[44] Olichney JM, Hansen LA, Hofstetter CR, Grundman M, Katzman R, Thal LJ (1995). Cerebral infarction in Alzheimer’s disease is associated with severe amyloid Angiopathy and hypertension. Arch Neurol.

[45] Prelli F, Castano E, Glenner GG, Frangione B (1988). Differences between vascular and plaque Core amyloid in Alzheimer’s disease. J Neurochem.

[46] Quon D, Wang Y, Catalano R, Scardina JM, Murakami K, Cordell B (1991). Formation of β-amyloid protein deposits in brains of transgenic mice. Nature.

[47] Radde R, Bolmont T, Kaeser SA, Coomaraswamy J, Lindau D, Stoltze L, Calhoun ME, Jäggi F, Wolburg H, Gengler S, Haass C, Ghetti B, Czech C, Hölscher C, Mathews PM, Jucker M (2006). Aβ42-driven cerebral amyloidosis in transgenic mice reveals early and robust pathology. EMBO Rep.

[48] Ringman JM, Monsell S, Ng DW, Zhou Y, Nguyen A, Coppola G, Van Berlo V, Mendez MF, Tung S, Weintraub S, Mesulam MM, Bigio EH, Gitelman DR, Fisher-Hubbard AO, Albin RL, Vinters HV (2016). Neuropathology of autosomal dominant Alzheimer disease in the national Alzheimer coordinating center database. J Neuropathol Exp Neurol.

[49] Rockenstein E, Mallory M, Mante M, Sisk A, Masliaha E (2001). Early formation of mature amyloid-beta protein deposits in a mutant APP transgenic model depends on levels of Abeta(1-42). J Neurosci Res.

[50] Scahill RI, Ridgway GR, Bartlett JW, Barnes J, Ryan NS, Mead S, Beck J, Clarkson MJ, Crutch SJ, Schott JM, Ourselin S, Warren JD, Hardy J, Rossor MN, Fox NC (2013). Genetic influences on atrophy patterns in familial Alzheimer’s disease: a comparison of APP and PSEN1 mutations. J Alzheimers Dis.

[51] Shinkai Y, Yoshimura M, Ito Y, Odaka A, Suzuki N, Yanagisawa K, Ihara Y (1995). Amyloid β-proteins 1—40 and 1—42(43) in the soluble fraction of extra- and intracranial blood vessels. Ann Neurol.

[52] Sim RB, Dodds AW, Mitc DA, Reid KBM, Sc J (2014). Early-onset amyloid deposition and cognitive deficits in transgenic mice expressing a double mutant form of amyloid precursor protein 695*. J Biol Chem.

[53] Sorbi S, Nacmias B, Forleo P, Piacentini S, Latorraca S, Amaducci L (1995). Epistatic effect of APP717 mutation and apolipoprotein E genotype in familial Alzheimer’s disease. Ann Neurol.

[54] Sturchler-Pierrat C, Abramowski D, Duke M, Wiederhold KH, Mistl C, Rothacher S, Ledermann B, Bürki K, Frey P, Paganetti PA, Waridel C, Calhoun ME, Jucker M, Probst A, Staufenbiel M, Sommer B (1997). Two amyloid precursor protein transgenic mouse models with Alzheimer disease-like pathology. Proc Natl Acad Sci U S A.

[55] Talarico G, Piscopo P, Gasparini M, Salati E, Pignatelli M, Pietracupa S, Malvezzi-Campeggi L, Crestini A, Boschi S, Lenzi GL, Confaloni A, Bruno G (2010). The London APP mutation (Val717Ile) associated with early shifting abilities and behavioral changes in two Italian families with early-onset Alzheimer’s disease. Dement Geriatr Cogn Disord.

[56] Tang MX, Stern Y, Marder K, Bell K, Gurland B, Lantigua R, Andrews H, Feng L, Tycko B, Mayeux R (1998). The APOE-epsilon4 allele and the risk of Alzheimer disease among African Americans, whites, and Hispanics. JAMA.

[57] Thal DR, Ghebremedhin E, Orantes M, Wiestler OD (2003). Vascular pathology in Alzheimer disease: correlation of cerebral amyloid Angiopathy and arteriosclerosis/Lipohyalinosis with cognitive decline. J Neuropathol Exp Neurol.

[58] Thal DR, Ghebremedhin E, Rüb U, Yamaguchi H, Del Tredici K, Braak H (2002). Two types of sporadic cerebral amyloid angiopathy. J Neuropathol Exp Neurol.

[59] Thal DR, Papassotiropoulos A, Saido TC, Griffin WS, Mrak RE, Kölsch H, Del Tredici K, Attems J, Ghebremedhin E (2010). Capillary cerebral amyloid angiopathy identifies a distinct APOE epsilon4-associated subtype of sporadic Alzheimer’s disease. Acta Neuropathol.

[60] Thomas AJ, Morris CM, Ferrier IN, Kalaria RN (2000). Distribution of amyloid beta42 in relation to the cerebral microvasculature in an elderly cohort with Alzheimer’s disease. Ann N Y Acad Sci.

[61] Vonsattel JPG, Myers RH, Tessa Hedley-Whyte E, Ropper AH, Bird ED, Richardson EP (1991). Cerebral amyloid angiopathy without and with cerebral hemorrhages: a comparative histological study. Ann Neurol.

[62] Waxman EA, Giasson BI (2008). Specificity and regulation of casein kinase-mediated phosphorylation of alpha-synuclein. J Neuropathol Exp Neurol.

[63] Zhang G, Xie Y, Wang W, Feng X, Jia J (2017). Clinical characterization of an APP mutation (V717I) in five Han Chinese families with early-onset Alzheimer’s disease. J Neurol Sci.

