# Supplementary material for: Prominent amyloid plaque pathology and cerebral amyloid angiopathy in APP V717I (London) carrier – phenotypic variability in autosomal dominant Alzheimer’s disease
Source: Acta Neuropathol Commun. 2020 Mar 12;8:31. doi: 10.1186/s40478-020-0891-3 (PMC7068954; doi:10.1186/s40478-020-0891-3)
Supplement: Supplementary file 1 — Additional file 1. Materials and Methods [12, 13, 31, 35, 62] [file 40478_2020_891_MOESM1_ESM.docx]

**Supplementary Information**

**Materials and Methods**

*Enrollment and tissue dissection*

The patient consented to brain donation to the University of Florida Neuromedicine Human Brain Tissue Bank (UF HBTB) and neuropathological workup in the Center for Translational Research in Neurodegenerative Disease (CTRND) according to protocols approved by the institutional review board of the University of Florida. Upon procurement the brain specimen consisting of cerebrum in continuity with brainstem and cerebellum was hemisected along a mid-sagittal plane. The left hemi-section was preserved in 10% buffered formaldehyde, and the right hemi-section was frozen for future ancillary studies. After fixation, the following areas were sectioned from the left brain and submitted for histologic study as paraffin-embedded blocks: left mid frontal gyrus, left orbital frontal gyrus, left cingulate gyrus, left striatum, left primary motor cortex, left lentiform nucleus, left superior/middle temporal gyrus, left amygdala, left primary sensory cortex, left sub-thalamic nucleus, left hippocampus, left angular gyrus, left primary visual cortex, left midbrain, left pons, left cerebellum, left medulla, and left cervical spinal cord.

*Genetic analysis*

DNA from fresh frozen tissue from the right brain was submitted to a directed Sanger sequencing panel, which screened for mutations in *APP* Exons 16 and 17, and all protein-coding exons of *PSEN1* and *PSEN2*. DNA from fresh frozen tissue was also submitted for *APOE* genotyping.

*Histology and Immunohistochemistry*

All sections derived from the paraffin-embedded blocks were stained with hematoxylin and eosin (H&E). Immunostaining of the sections was performed using standard methods [13]. Unless otherwise indicated, tissue sections were rehydrated and heat-induced epitope retrieval (HIER) was performed in a steam bath for 60 min in a 0.05% Tween-20 solution. Endogenous peroxidase was quenched by incubation of sections in 1.5% hydrogen peroxide/0.005% Triton-X-100 diluted in pH 7.4 sterile phosphate buffered saline (PBS) (Invitrogen) for 20 min.

Non-specific antibody binding was minimized with a 2% fetal bovine serum (FBS)/0.1M Tris, pH 7.6 blocking solution; primary antibodies were diluted in blocking solution and applied to tissue sections at 4°C overnight. Biotinylated secondary antibody (Vector Laboratories; Burlingame, CA) was diluted in blocking solution (1:3000) and applied to sections for 1 h at room temperature (RT). To enhance detection of the immunocomplexes, an avidin-biotin complex (ABC) system (Vectastain ABC Elite kit; Vector Laboratories, Burlingame, CA) was utilized in conjunction with chromogen 3,3′-diaminobenzidine (DAB kit; KPL, Gaithersburg, MD), allowing for observation of immunocomplexes. Counterstaining was performed using Mayer’s hematoxylin (Sigma Aldrich, St. Louis, MO). For Aβ immunohistochemistry, epitope unmasking was enhanced by treating sections with 70% FA for 10 min at RT prior to HIER incubation. An optimized antigen retrieval solution of 0.05% Tween-20 in modified citrate buffer (Target Retrieval Solution Citrate pH 6; Agilent, Santa Clara, CA) was used for HIER and sections were allowed to cool to RT after 30 min incubation in the steam bath. Sections were rinsed in 0.1M Tris, pH 7.6 and the blocking solution was composed of 2% FBS/0.1M Tris, pH 7.6. Primary antibodies were diluted in 0.1M Tris, pH 7.6 (4G8 1:1000; 12F4 1:500; 13.1.1 1:800) and applied to tissue sections at 4 °C overnight. Impress polymer secondary antibody (Vector Laboratories; Burlingame, CA) was applied to sections for 90 min at RT and DAB solution was warmed to 37°C prior to application.

For TDP43 immunohistochemistry, epitope unmasking was enhanced by immersing sections into 70% formic acid (FA) for 30 min at RT prior to HIER incubation described above. Immunohistochemistry for tau was performed, utilizing antibodies directed toward phospho-epitopes pSer199/pSer202/pThr205 (AT8, Invitrogen, Carlsbad, CA). Immunohistochemistry for Aβ was performed utilizing antibodies directed toward a mid-domain epitope (4G8, Biolegend, San Diego, CA), a C-terminal epitope specific for Aβ_1-40_ (13.1.1 [40.1] [31]), a gift from Todd E. Golde), and a C-terminal epitope specific for Aβ_1-42_ (12F4, EMD Millipore Corporation, Temecula, CA). Immunohistochemistry for alpha-synuclein was performed, utilizing antibodies directed toward phospho-epitope pSer129 (81A [62]) and amino acid residues 130-140 (94-3A10 [12]). TDP-43 immunohistochemistry was used utilizing TARDBP (Proteinech, Manchester, UK), an antibody directed toward pan-TDP-43 (N-terminal epitope, recognizes full length, posttranslationally modified and truncated forms of TDP-43). See supplementary table 1 for details.

*Image acquisition*

Slides were digitally scanned using Aperio ScanScope CS instrument (40× magnification; Aperio Technologies Inc., Vista, CA), and images of representative areas of pathology were captured using the ImageScope software (40× magnification; Aperio Technologies Inc.).

*Neuropathologic assessment*

Staging of AD neuropathologic changes, including semi-quantitative assessment of neuritic plaque burden by immunohistochemistry, was conducted per guidelines published by the National Institute on Aging-Alzheimer’s Association [35] by JTL, ATY and SP.
