# Supplementary material for: Prominent amyloid plaque pathology and cerebral amyloid angiopathy in APP V717I (London) carrier – phenotypic variability in autosomal dominant Alzheimer’s disease
Source: Acta Neuropathol Commun. 2020 Mar 12;8:31. doi: 10.1186/s40478-020-0891-3 (PMC7068954; doi:10.1186/s40478-020-0891-3)
Supplement: Supplementary file 2 — Additional file 2: Table S1. List of Antibodies used for this study [file 40478_2020_891_MOESM2_ESM.docx]

**Supplementary table 1**

**List of Antibodies used for this study**

| **Group** | **Name** | **Epitope** | **Source** | **Concentration** | **Pretreatment** |
| --- | --- | --- | --- | --- | --- |
| APP/Aβ | 4G8 | Amyloid-beta_17-24_ | Biolegend, San Diego, CA | 1:1000 | FA (10min) |
|  | 12F4 | Amyloid-beta_1-42_ | EMD Millipore Corporation, Temecula, CA | 1:500 | FA (10min) |
|  | 13.1.1 (Ab40.1) | Amyloid-beta_1-40_ | Todd Golde | 1:800 | FA (10min) |
| TDP43 | #10782-2-AP | N-terminal epitope, recognizes full length, post-translationally modified and truncated forms of TDP43 | Proteintech, Rosement, Il | 1:1000 | FA (30min) |
| α-synuclein | 81A | pSer129 | Benoit Giasson | 1:3000 | None |
| α-synuclein | 94-3A10 | α-synuclein (aa. 130-140) | Benoit Giasson | 1:10,000 | None |
