# Supplementary material for: Prominent amyloid plaque pathology and cerebral amyloid angiopathy in APP V717I (London) carrier – phenotypic variability in autosomal dominant Alzheimer’s disease
Source: Acta Neuropathol Commun. 2020 Mar 12;8:31. doi: 10.1186/s40478-020-0891-3 (PMC7068954; doi:10.1186/s40478-020-0891-3)
Supplement: Supplementary file 3 — Additional file 3:Table S2. Summary of patient samples used in this study [35, 41, 61] [file 40478_2020_891_MOESM3_ESM.docx]

**Supplementary table 2**

**Summary of patient samples used in this study**

| Case | Age | Gender | APOE | Braak stage ^1^ | NFT score^1^ | Aβ phase^1^ | Aβ score^1^ | CERAD ^1^ | CERAD score^1^ | CAA grade^2^ | Other pathology |
| --- | --- | --- | --- | --- | --- | --- | --- | --- | --- | --- | --- |
| ADAD | 66 | female | e2/e3 | VI | B3 | 5 | A3 | frequent | C3 | severe | LATE stage 2^3^ |
| SAD1 | 68 | male | e3/e3 | IV | B2 | 4 | A3 | frequent | C3 | moderate | None |
| SAD2 | 83 | male | e4/e4 | VI | B3 | 5 | A3 | frequent | C3 | severe | None |

^1^ 2012 NIA-AA criteria [35]

^2^ [61]

^3^ [41]
